# Recent advances and current limitations of available technology to optically manipulate and observe cardiac electrophysiology

**DOI:** 10.1007/s00424-023-02858-0

**Published:** 2023-09-28

**Authors:** Gerard A. Marchal, Valentina Biasci, Ping Yan, Chiara Palandri, Marina Campione, Elisabetta Cerbai, Leslie M. Loew, Leonardo Sacconi

**Affiliations:** 1 Institute of Clinical Physiology (IFC-CNR), Florence, Italy; 2 European Laboratory for Non-Linear Spectroscopy-LENS, Sesto Fiorentino, Florence, Italy; 3 Department of Experimental and Clinical Medicine, University of Florence, Florence, Italy; 4 R. D. Berlin Center for Cell Analysis and Modeling, University of Connecticut School of Medicine, Farmington, CT USA; 5 Department NeuroFarBa, University of Florence, Florence, Italy; 6 Institute of Neuroscience (IN-CNR) and Department of Biomedical Science, University of Padua, Padua, Italy; 7 Institute for Experimental Cardiovascular Medicine, University Heart Center and Medical Faculty, University of Freiburg, Freiburg, Germany

**Keywords:** Optogenetics, Calcium imaging, Optical mapping, All-optical, Spectral congestion

## Abstract

Optogenetics, utilising light-reactive proteins to manipulate tissue activity, are a relatively novel approach in the field of cardiac electrophysiology. We here provide an overview of light-activated transmembrane channels (optogenetic actuators) currently applied in strategies to modulate cardiac activity, as well as newly developed variants yet to be implemented in the heart. In addition, we touch upon genetically encoded indicators (optogenetic sensors) and fluorescent dyes to monitor tissue activity, including cardiac transmembrane potential and ion homeostasis. The combination of the two allows for all-optical approaches to monitor and manipulate the heart without any physical contact. However, spectral congestion poses a major obstacle, arising due to the overlap of excitation/activation and emission spectra of various optogenetic proteins and/or fluorescent dyes, resulting in optical crosstalk. Therefore, optogenetic proteins and fluorescent dyes should be carefully selected to avoid optical crosstalk and consequent disruptions in readouts and/or cellular activity. We here present a novel approach to simultaneously monitor transmembrane potential and cytosolic calcium, while also performing optogenetic manipulation. For this, we used the novel voltage-sensitive dye ElectroFluor 730p and the cytosolic calcium indicator X-Rhod-1 in mouse hearts expressing channelrhodopsin-2 (ChR2). By exploiting the isosbestic point of ElectroFluor 730p and avoiding the ChR2 activation spectrum, we here introduce a novel optical imaging and manipulation approach with minimal crosstalk. Future developments in both optogenetic proteins and fluorescent dyes will allow for additional and more optimised strategies, promising a bright future for all-optical approaches in the field of cardiac electrophysiology.

**Supplementary Information:**

The online version contains supplementary material available at 10.1007/s00424-023-02858-0.

## Introduction

“Optogenetics” is a term introduced by Deisseroth and colleagues [13], covering the implementation of light-sensitive proteins. Since the discovery of light-sensitive bacteriorhodopsins in the 1970s and the implementation of optogenetics in neurobiology in the early 2000s, optogenetics have relatively recently been introduced in cardiac research. Optogenetic actuators are photoreactive proteins which impact cell function in a light-dependent manner and have been proven to be an especially powerful tool due to the ability to target specific tissue types and sites [9]. This high degree of specificity is the consequence of two factors: (i) the possibility to target specific cell types to express optogenetic proteins, and (ii) the ability to apply stimulatory illumination to a specific area. This high level of control is also of particular interest for applications in the heart, as it allows for the targeting of specific cell types (e.g. cardiomyocytes, fibroblasts) as well as selected regions in the myocardium. In addition to optogenetic actuators, genetically encoded indicators—which have also been termed optogenetic sensors—have been developed. These sensors allow for the monitoring of membrane voltage and intracellular ion concentrations without the use of fluorescent dyes. Hence, the field of optogenetics opens a range of possibilities, both for manipulating and monitoring of cellular and organ function.

### All-optical approaches

An exciting consequence from the development of optical actuators and sensors is the emergence of all-optical approaches, allowing for completely contact-free approaches to simultaneously monitor and manipulate cell and tissue activity. All-optical approaches promise increased flexibility in studying arrhythmogenesis in whole hearts, as well as drug testing and development of personalised medicine when employing cellular systems. As such, the implementation of optogenetics promises a plethora of opportunities in studying and modulating cardiac electrophysiology. However, these opportunities are accompanied by various challenges which currently hamper full utilisation of optogenetic approaches. One major challenge is the rise of spectral congestion, caused by the overlap of excitation/activation and emission spectra of various optogenetic proteins and/or fluorescent dyes [45]. In this review, we will provide an overview of current advancements in optogenetics, as well as currently remaining obstacles remaining to be tackled to fully exploit this enlightening technique.

## Optogenetic actuators

Various types of optogenetic actuators have been employed in neurobiological studies, inducing light-triggered modulation of protein activity, subcellular localisation of proteins, and protein interactions, in addition to signalling processes and neurone firing [8]. Among the different types of photoreactive proteins, microbial opsins are transmembrane channels which allow ions to cross the membrane, generating—depending on the type of opsin—a depolarising (excitatory) or hyperpolarising/repolarising (inhibitory) current [12]. Hence, optogenetic strategies can be applied to modulate a wide range of cellular functions, including electrophysiological properties. Various optogenetic actuators have been introduced and characterised, including non-selective cation channels, as well as channels specifically generating an inward Ca^2+^ current [26] or an outward H^+^ current [36]. Apart from the content and direction of the current generated, other factors characterising the various opsins and determining their applicability for (cardiac) functional modulation include (i) peak activation spectral wavelength, (ii) width of the activation spectrum, (iii) amplitude of the current generated, and (iv) speed of channel activation and inactivation.

### Channelrhodopsin-2

**Fig. 1:**
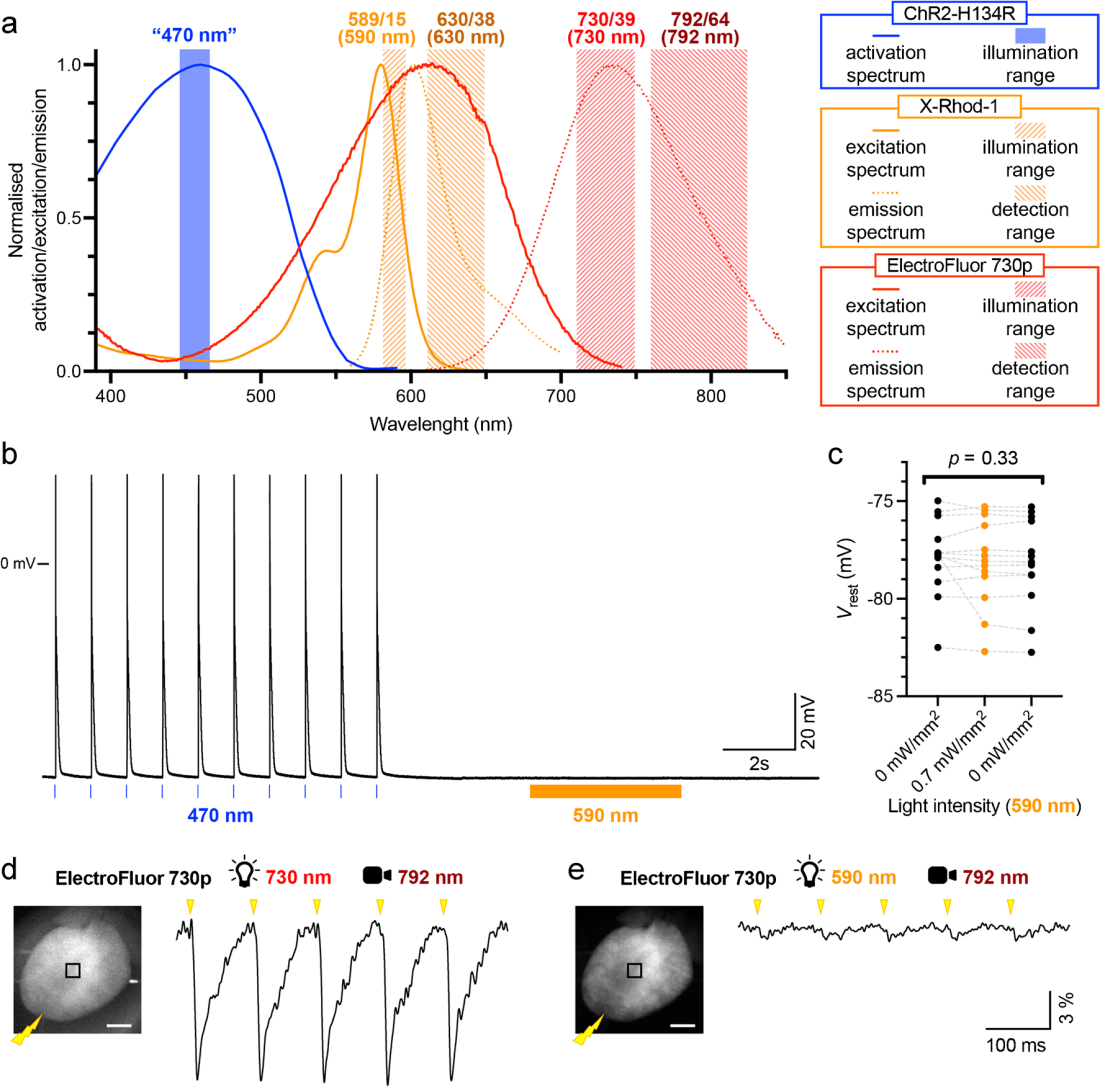
Crosstalk-free imaging of cytosolic calcium (Ca_i_^2+^) through X-Rhod-1 and transmembrane potential though the voltage-sensitive dye (VSD) ElectroFluor 730p. **a** Normalised activation spectrum of H134R-channelrhodopsin-2 (ChR2-H134R), excitation and emission spectra of the Ca_i_^2+^ indicator X-Rhod-1 and VSD ElectroFluor 730p, with the ranges of illumination and detection indicated. **b** Representative patch-clamp transmembrane potential trace recorded in an isolated ChR2-expressing murine ventricular cardiomyocyte. Optogenetic excitability was confirmed inducing action potentials by blue light pulses (470 nm, 3 ms; blue lines). Amber light (590 nm, orange) was then applied to assess the impact of X-Rhod-1 excitation light on resting membrane potential (*V*_rest_). **c** The average *V*_*r*est_ in individual ChR2-expressing murine ventricular cardiomyocytes before, upon, and after illumination at 590 nm. No significant impact of 590 nm illumination on *V*_rest_ was detected. **d**, **e** *Left*: representative fluorescence images (F0) of a mouse heart stained with ElectroFluor 730p, with centred excitation (lamp icon) and detection (camera icon) wavelength. Hearts were electrically stimulated at the apex (yellow bolt) at 10 Hz. *Right*: fluorescent signals (ΔF/F) extracted from the central portion of the ventricle during electrical stimulation (yellow triangles). While exciting ElectroFluor 730p at 730 nm resulted in clearly distinguishable inverse action potentials (**d**), variations in transmembrane potential caused minimal signal deflections when exciting at 590 nm (**e**). Data in panel (**c**) was collected in 13 cardiomyocytes from 2 mice, with variance tested by a one-way ANOVA. Scale bar: 2 mm (**d**, **e**)

Among the optogenetic actuators, the opsin channelrhodopsin-2 (ChR2), and specifically the ChR2-H134R variant, which generates a current with a larger amplitude [37], has been most widely implemented in cardiac research [5, 6, 11, 39]. This non-selective cation-specific transmembrane channel allows positively charged ions to enter the cell when illuminated with blue light (± 470 nm, Fig. 1a), thereby generating a depolarising current [37]. In the heart, ChR2 has been utilised for optical pacing, where the channel is activated by a high-intensity illumination, generating a current which amplitude is enough to trigger action potentials (APs) and induce atrial and/or ventricular activation [1, 5, 32]. In addition to pacing, this approach has also been employed for termination of arrhythmias in the ventricle and the atria [1, 6, 11, 32, 38, 47], introducing an optical method for cardioversion which is more energy-efficient and less burdensome than traditional lead-based approaches to achieve defibrillation. In addition to pacing and defibrillation, the ChR2 has been implemented to correct short and long QT phenotypes in isolated cardiomyocytes. This was accomplished by the delivery of a timed, high-intensity pulse of blue light during either the early or late repolarisation phase, resulting in an abbreviation and prolongation of AP duration, respectively [18]. Hence, stimulation of ChR2 by high-intensity light can be utilised to attain cardiac pacing and defibrillation, as well as the modulation of AP characteristics.

Apart from these approaches employing supra-threshold illumination, recent studies uncovered the possibility to modulate cardiac electrophysiological dynamics by the application of sub-threshold illumination. This approach leads to the generation of a depolarising current, which is not sufficient to trigger APs, but does modulate AP characteristics. By the use of patterned sub-threshold illumination, intraventricular gradients in cardiac activation and repolarisation kinetics could be generated, with conduction slowing and delayed repolarisation specifically in the illuminated area [34]. Moreover, application of sub-threshold illumination at the bulk of the ventricular surface results in the emergence of alternans in AP upstroke and repolarisation. Surprisingly, this rise of cardiac alternans promoted the spontaneous termination of induced ventricular tachycardia [4]. Therefore, apart from high-intensity stimulation of ChR2, sub-threshold stimulation can be applied to modulate cardiac activation and repolarisation kinetics in a spatially specific manner, as well cardiac alternans. These approaches can be highly relevant for investigations regarding arrhythmogenic mechanisms in the setting of heterogeneities in conduction and/or repolarisation, as well as mechanisms underlying alternans and termination of arrhythmias.

### Spectrum-shifted channelrhodopsins

In addition to “classic” ChR2-H134R, various variants of ChR which exhibit an activation spectrum which is shifted either to the blue or the red spectrum have recently been introduced. This shift in the peak activation wavelength potentially allows for combinations with fluorescent dyes and other reporters excited by light in the spectral range which would activate conventional ChR2, circumventing spectral congestion. Blue-shifted variants includes CheRiff which has an only slightly blue-shifted peak activation wavelength (± 460 nm), but has a more narrow activation spectrum, faster activation and inactivation kinetics, and higher current amplitude as compared to ChR2-H134R [19]. However, CheRiff has not yet been utilised in cardiac research, potentially due to the spectral similarities to ChR2 and the abundance of ChR2-expressing models.

Crucially, the use of red stimulation light instead of blue light results in deeper tissue penetration of illumination, as tissue penetration of blue light is not optimal [59]. Therefore, optogenetic ventricular defibrillation approaches are theoretically more efficient when red light-sensitive ion channels would be used, as well as allowing for lower-energy approaches [24, 46]. However, various red-shifted ChR variants have presented significant deficiencies hampering optimal implementation in the heart. Red-shifted ChR variants currently used in cardiac pacing and defibrillation approaches include red activatable channelrhodopsin (ReaChR) and ChRmine [20, 39–42], which exhibit a peak activation wavelength of ± 610 nm and ± 540 nm, respectively [33, 52]. Despite their red-shifted peak activation wavelength, both ReaChR and ChRmine exhibit a wide activation spectrum [33, 52], indicating that these channels would also be activated by blue light. Indeed, cardiac pacing has been achieved by activating ReaChR with blue light (470 nm) [41]. Hence, the wide activation spectra of these red-shifted ChR variants play into the rise of spectral congestion, hampering combinations with fluorescent dyes or optogenetic sensors. Moreover, ChRmine has been shown to inactivate extremely slow, with a current decay time about 6 times longer than ChR2 [52]. This slow inactivation aspect causes the rise of a “persistent” current, limiting the temporal specificity optical stimulation and rendering it not ideal for cardiac implementations.

Apart from these red-shifted ChR variants already implemented in cardiac research, novel variants have been introduced recently, improving channel kinetics or further shifting the peak activation wavelength. Recent introductions include bReaChES, Chrimson variants, and ChroME2.0 opsins [25, 44, 52, 58]. However, these channels still suffer from a wide activation spectrum and/or slow inactivation kinetics [48, 52, 53], limiting their applicability for cardiac research. Taken together, while these red-shifted ChRs do benefit from the deeper penetration of red light in cardiac tissue, combinations with proteins and compound stimulated by blue light and wavelengths above are still not possible. The development of variants with high current amplitude, fast inactivation, and narrow activation spectra would aid preventing spectral congestion and temporal specificity of optogenetic stimulation.

### Inhibitory optogenetic actuators

Apart from the depolarising current generating ChRs described above, inhibitory opsins are also of high interest for cardiac studies. These channels provide, depending on the timing of optogenetic stimulation, a hyperpolarising current inhibiting excitability or a repolarising current shortening AP duration. Various classes of rhodopsins generating this effect are available, including modified ChRs and halorhodopsins pumps which generate an inward Cl^−^ current, and archearhodopsins which transport ions (typically H^+^) towards the extracellular space. From the latter class, archaerhodopsin-3 (Arch) is able to virtually completely silence neurons [10] and has also been proven effective in neonatal rat ventricular cardiomyocytes [17]. However, archearhodopsins may have to be avoided in cardiac approaches since the movement of ions from the intra- to the extracellular space lowers extracellular pH [60], which impacts cardiomyocyte function [21]. Meanwhile, the anion (Cl^−^) conducting channelrhodopsin-1 derived from *Guillardia theta* (GtACR1) has been proven more efficient in neonatal rat ventricular cardiomyocytes, generating a larger current amplitude, being more effective in silencing spontaneous electrical activity and shortening AP duration [17]. In addition, anion channelrhodopsin-2 (ACR2) has also been successfully introduced in human induced pluripotent stem cell-derived cardiomyocytes to suppress cardiomyocyte excitability and correct a long QT phenotype [18].

Moreover, although inhibitory optogenetic channels have not yet been implemented in whole-heart models, computational studies have highlighted the capability of GtACR1 to terminate atrial and ventricular re-entry based arrhythmias [43]. Crucially, this study suggests that the energy required for optogenetic defibrillation is several orders of magnitude lower than when using ChR2. Hence inhibitory optogenetic channels are highly promising for cardiac defibrillation and AP duration shortening purposes, which could be a novel approach in long QT syndromes. However, their applicability in whole hearts is yet to be proven.

## Optogenetic sensors

Besides optogenetic actuators, which affect cell function in a light-dependent manner, optogenetics can also be implemented to monitor cell activity through optogenetic sensors, also known as genetically encoded indicators. Various classes of optogenetic sensors allow for sensing of intracellular ion concentrations, activity of signal transductors, or transmembrane potential (voltage) in order to visualise action potentials (APs) [27]. Indeed, the optogenetic voltage indicator VSFP2.3 has already been applied in single cardiomyocytes and whole murine hearts [7], while archon1 has been used in single hiPSC-derived cardiomyocytes and engineered heart tissue [51]. Moreover, the applicability of the dual optogenetic sensing has been demonstrated implementing the voltage indicator chimeric VSFP-butterfly CY and calcium indicator GcaMP6f in zebrafish hearts [54]. This approach allowed for monitoring of the spatiotemporal characteristics of cardiac activation and repolarisation, local AP characteristics, and calcium homeostasis in vivo in embryonic and juvenile zebrafish.

However, as described in depth in previous reviews, various issues need to be tackled to be able to fully abolish the need of fluorescent dyes as voltage indicators [14, 45]. Specifically, optogenetic voltage indicators typically either lack rapid kinetics, thereby failing to correctly report the AP upstroke, or display inadequate sensitivity hampering detection of subtle changes in transmembrane potential [2, 51]. Still, these developments emphasise the promise of optogenetic sensors and pave the way for novel, all-optical approached without the use of fluorescent dyes. Until optogenetic sensors can accurately depict variations in voltage and ion homeostasis, fluorescent dyes remain an excellent alternative to measure electrophysiological and ion handling ex vivo.

## Fluorescent dyes

As mentioned above, while all-optical approaches utilising both optogenetic actuators and sensors are a highly promising, fundamental issues still stand in the way of making full use of combinations of these optogenetic tools. One of the central problems to be tackled is spectral congestion leading to optical crosstalk, where the activation or emission wavelength of one protein will affect another. Given the present limitations of both optogenetic actuators and sensors, fluorescent dyes are currently preferable for optical detection of transmembrane potential (voltage) and intracellular ion concentration in whole hearts expressing optogenetic actuators. The abundance of fluorescent dyes available and the continuous development of novel dyes allows for the selection of combinations of multiple fluorescent dyes, while applying optogenetic stimulation, with minimal crosstalk.

For ex vivo cardiac optical mapping, two main classes of synthetic, fast fluorescent dyes are commonly used. Potentiometric voltage-sensitive dyes (VSDs) responding to transmembrane potential can be used to map AP propagation and repolarisation timing across the heart. On the other hand, cytosolic calcium (Ca_i_^2+^) indicators are applied to assess tissue-wide intracellular Ca^2+^ handling and Ca^2+^ transients. Transmembrane potential and calcium handling are considered two of the most important parameters of cardiac electrophysiology, and their interplay is crucial for normal cardiac function through excitation–contraction coupling [3]. Therefore, these two classes of fluorescent dyes are frequently used simultaneously for multi-parametric imaging, providing essential insights into the interplay of transmembrane potential and Ca_i_^2+^, especially in the setting of pathological conditions to study arrhythmia mechanisms [16, 22, 28, 50]. Commonly used VSDs include Di-4-ANEPPS and Di-8-ANEPPS, which were developed by Loew and colleagues [15]. These dyes are not particularly suitable in combination with optogenetic approaches since the blue–green excitation light (510–530 nm) overlaps with the activation spectrum ChR2 [37] and is heavily affected by light scattering in cardiac tissue [59]. To overcome these limitations, the red-shifted (640 nm) potentiometric dye di-4-ANBDQPQ has been recently introduced for ex vivo cardiac optical mapping [30, 35]. This dye allows for combinations with either optogenetic actuators including ChR2 [4, 34], or popular Ca_i_^2+^ indicators including rhod-2 [28], but not in combination due to spectral overlap.

Recent efforts have focussed on the development of potentiometric dyes this near-infrared (NIR) window. Dyes operating in this window offer even further benefits as compared to red-shifted dyes due to the low light scattering and low absorption of (oxy)haemoglobin and water in the 650–900 nm range [55]. Recently, Yan and collaborators synthetised and characterised the novel VSD ElectroFluor 730p, which is excitable in the NIR region of the spectrum [57]. Since the excitation spectrum of this VSD is far removed from the ChR2 activation spectrum, this would allow novel combinations with other indicators operating in the window in between.

## Exploring new possibilities of multi-parameter mapping and optogenetic stimulation

To explore the new possibilities offered by the novel VSD ElectroFluor 730p, we here combine optical calcium and voltage mapping with optogenetic stimulation in mouse hearts expressing ChR2-H134R. In Fig. 1a, the activation spectrum of ChR2 and the excitation and emission spectra of ElectroFluor 730p and the Ca_i_^2+^ indicator X-Rhod-1 are shown. In addition, the ranges of ChR2 stimulation light, as well as the ranges of excitation light and emission light detection of both fluorescent dyes, are indicated. A full description of the light sources and filters used can be found in the Supplemental Material.

The selected excitation wavelength for X-Rhod-1 (± 590 nm) did not activate ChR2, as high-intensity light pulses at this wavelength did not induce APs or modify resting membrane potential in isolated ChR2-expressing cardiomyocytes (Fig. 1b, c). As expected, electrical stimulation at the apex caused evident deflections in fluorescent signal centred at 792 nm when exciting at 730 nm, confirming the ability of ElectroFluor 730p to detect APs (Fig. 1d). By contrast, no considerable fluctuation of fluorescence at 792 nm was detected upon exciting the VSD at 590 nm (Fig. 1e). Hence, the excitation light used for the Ca_i_^2+^ indicator X-Rhod-1 does not induce fluctuations in the emission of the VSD ElectroFluor 730p. This is due to the proximity of the 590-nm excitation light to the isosbestic point of the ElectroFluor 730p excitation spectrum [57], resulting in minimal differences in emission upon changes in transmembrane potential. Therefore, the excitation wavelengths selected for each dye do not affect emission of the other, minimising optical crosstalk.

**Fig. 2:**
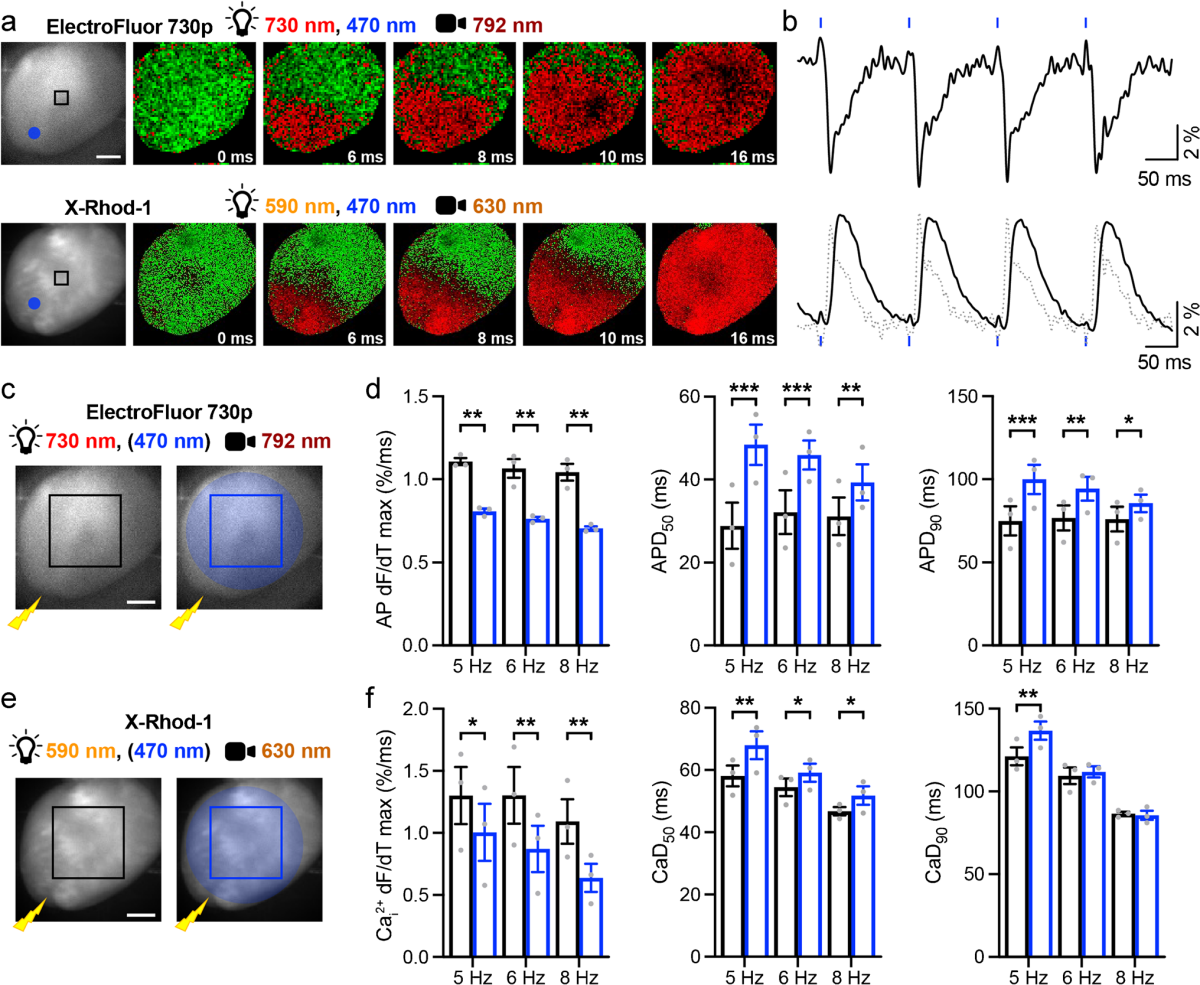
Characterisation of optical transmembrane potential and cytosolic calcium (Ca_i_^2+^) mapping and optogenetic manipulation in channelrhodopsin-2 (ChR2)-expressing mouse hearts. **a** Representative F0 (*left*) and five ΔF/F (*right*) fluorescence images of a ChR2-expressing mouse heart stained with both the voltage-sensitive dye ElectroFluor 730p and Ca_i_^2+^ indicator X-Rhod-1. The relevant centred excitation (lamp icon) and detection (camera icon) wavelength are indicated above the images. The heart was paced at the apex by a single-point illumination pattern (blue circle) at a frequency of 7 Hz (3 ms pulse duration). The propagation of the action potential (AP, top) and Ca_i_^2+^ (bottom) wavefront, with diastolic tissue in green and excited tissue in red can be observed in the ΔF/F images. **b** Fluorescent traces (ΔF/F) extracted from the central portion of the ventricle, showing the transmembrane potential (ElectroFluor 730 signal, top) and Ca_i_^2+^ cycle (X-Rhod-1 signal, bottom). The Ca_i_^2+^ trace also includes an inverted transmembrane potential trace (grey, dotted line) to indicate the delay between AP upstroke and Ca^2+^ release. The timing of optogenetic stimulation is indicated by blue lines. **c**, **e** Representative F0 fluorescence images of a ChR2-expressing mouse heart stained with both ElectroFluor 730p and X-Rhod-1, with the relevant centred excitation (lamp icon) and detection (camera icon) light wavelength indicated. Hearts were electrically stimulated at the apex (yellow bolt) and measured at baseline or during optogenetic stimulation with blue light at a sub-threshold intensity (illuminated area indicated in transparent blue). **d** Maximum action AP upstroke velocity (AP dF/dT max) and AP duration at 50% and 90% repolarisation (APD_50_, APD_90_) in the absence (black) and presence (blue) of sub-threshold illumination at pacing frequencies of 5, 6, and 8 Hz. **f** Maximum Ca_i_^2+^ upstroke velocity (Ca_i_^2+^ dF/dT max) and duration at 50% and 90% of Ca^2+^ extrusion (CaD_50_, CaD_90_) in the absence (black) and presence (blue) of sub-threshold illumination at pacing frequencies of 5, 6, and 8 Hz. Data was collected from the indicated central ventricular area in 3 hearts. *p < 0.05, **p < 0.01, ***p < 0.001 (two-way repeated measurements ANOVA with Tukey’s post-hoc test), scale bars: 2 mm

Next, we performed optical mapping in hearts loaded with both the ElectroFluor 730p and X-Rhod-1 dyes, while performing optogenetic stimulation at the apex of the heart. A clear activation pattern was visible for both the ElectroFluor 730p (voltage) and X-Rhod-1 (Ca_i_^2+^) signal, moving from the apex to the base (Fig. 2a). Also, fluorescent traces displayed distinct characteristics of APs and Ca_i_^2+^ transients (Fig. 2b). We subsequently tested whether the system is able to detect manipulation of electrophysiological characteristics induced by sub-threshold illumination (Fig. 2c, e). In line with our previous studies [4, 34], sub-threshold stimulation of ChR2 resulted in decreased AP upstroke velocity and prolonged repolarisation (Fig. 2d). Assessing the X-Rhod-1 signal, we observed a similar impact on Ca_i_^2+^ handling, indicating that sub-threshold illumination results in slowed Ca^2+^ release and extrusion kinetics (Fig. 2f). Overall, we here demonstrate the applicability of simultaneous voltage and Ca_i_^2+^ mapping, while also employing optogenetic stimulation.

## Discussion

We here provide an overview of the current state-of-the-art in cardiac optogenetics, covering optogenetic actuators and sensors, as well as selected fluorescent dyes. The current limitations of optogenetic sensors, as well as the issue of spectral congestion, currently restrain the possibility of full-optogenetic detection and manipulation models. However, as shown in the current study, novel developments in fluorescent dyes do open possibilities for all-optical, multi-parameter approaches. Taking advantage of the isosbestic point of ElectroFluor 730p, the excitation light for X-Rhod-1 distorts the voltage trace to a negligible extent, thereby allowing simultaneous assessment of both transmembrane potential and Ca_i_^2+^ when using these two specific fluorescent dyes. In addition, the distance to the ChR2 activation spectrum enables simultaneous application of optogenetic manipulation of cardiac electrophysiology. However, while this configuration allows near crosstalk-free multi-parameter mapping in murine hearts, the isosbestic point of VSDs varies among species due to the composition of the sarcolemma [28]. As such, this shift in isosbestic point should be carefully considered when implementing the presented approach in other species.

Although we here demonstrate the possibility of simultaneous whole-heart transmembrane potential and Ca_i_^2+^ mapping, we did not perform true multi-channel simultaneous mapping in the current study. Simultaneous mapping can be performed through a (low-cost) dual-camera setup [49], or with a single camera through either stroboscopic imaging or using a path splitter [23, 29]. In addition, it should be noted that the blue ChR2 stimulation light did cause a shift in baseline of both ElectroFluor 730p and X-Rhod-1, causing a deflection in both the voltage and Ca_i_^2+^ traces. While this did not affect measurements when applying continuous optical stimulation, computational approaches may improve this issue when administering pulsed optical stimulation.

Despite the technical limitations of the current study, we here demonstrate that current developments allow for exciting new possibilities with respect to all-optical mapping and manipulation approaches. Specifically, studying transmembrane potential and Ca_i_^2+^ in combination with ChR2 stimulation allows for further understanding and optimisation of a wide range of optogenetics-based strategies, including cardioversion/defibrillation approaches and the correction of electrophysiological defects through AP shaping.

Moreover, the approach introduced here is adaptable for in situ and in vivo approaches, as the fluorescent dyes used here are characterised by red-shifted excitation and emission spectra. These characteristics should allow for sufficient penetration in blood-perfused tissue, as a combination of a relatively more blue-shifted VSD and Ca_i_^2+^ indicator were previously successfully implemented in situ [29]. These in situ and in vivo optical mapping techniques previously described in rat and pig models, respectively [29, 31], allow for assessment of cardiac function in a setting where the cardiac coupling to the nervous and endocrine systems remain intact. Thereby, these approaches allow for measuring electrophysiological parameters in a native environment, providing further valuable insights in cardiac regulation and arrhythmia mechanisms.

Additionally, extending the concept of optogenetic manipulation with multi-parameter mapping to other physiological parameters offers intriguing new possibilities, including the monitoring of non-electrophysiological parameters. These opportunities are underscored by the previous demonstration of simultaneous mapping of transmembrane potential, Ca_i_^2+^, and the metabolic state marker NADH [28]. Moreover, optogenetic manipulation of cardiac function can potentially be extended to non-electrophysiological parameters, including reactive oxygen species [56].

Apart from the recent developments described in the current paper, forthcoming developments in optogenetic proteins as well as fluorescent probes will further extend opportunities to study and manipulate cardiac (patho)physiology. Together, these advances in imaging techniques, optogenetic proteins, and fluorescent dyes allow for a further dissection of cardiac physiology and pathophysiological processes, creating an exciting prospect for the future.

## Material and methods

All materials and methods can be found in the Supplementary Material.

### Supplementary Information

Below is the link to the electronic supplementary material.

Supplementary file1 (DOCX 25 KB)

## Data Availability

The data that support the findings of this study are available from the corresponding author upon reasonable request.
