# Supplementary material for: Recent advances and current limitations of available technology to optically manipulate and observe cardiac electrophysiology
Source: Pflugers Arch. 2023 Sep 28;475(11):1357–66. doi: 10.1007/s00424-023-02858-0 (PMC10567935; doi:10.1007/s00424-023-02858-0)
Supplement: Supplementary file 1 — Supplementary file1 (DOCX 25 KB) [file 424_2023_2858_MOESM1_ESM.docx]

Recent advances and current limitations of available technology to optically manipulate and observe cardiac electrophysiology

**- Supplemental Material -**

# Material and methods

## Mouse model and heart excision

The ChR2-*Myh6*-cre^+^ transgenic mice with cardiomyocyte-specific expression of H134R-ChR2 used in this study were generated as previously described [4]. All animal handling and experimental procedures were performed in accordance with the guidelines from Directive 2010/63/EU of the European Parliament on the protection of animals used for scientific purposes. The experimental protocol was approved by the Italian Ministry of Health (protocol number 531/2022-PR).

Mice aged 6 months old were heparinised by intraperitoneal injection (0.1 mL, 500 units) and euthanised by isoflurane inhalation overdose (5%), after which the heart was excised.

## Cardiomyocyte isolation and patch clamp

Enzymatic dissociation of ChR2 hearts to obtain single ventricular cardiomyocytes was performed as described previously [2]. Excised hearts were immediately bathed in a cell isolation buffer containing (in mM): 120 NaCl, 1.2 MgCl_2_, 10 KCl, 1.2 KH_2_PO_4_, 10 glucose, 10 HEPES, 20 taurine, 5 pyruvate (pH 7.4; NaOH). Next, the aorta was canulated and perfused with cell isolation buffer (oxygenated) for 15 minutes at 36 °C at a constant flow of 3 mL/min. Subsequently, the heart was perfused for 8 minutes with cell isolation buffer supplemented with 0.1 mg/mL Liberase TM (Roche Applied Sciences, Penzberg, DE). Then, the ventricular tissue was cut into small pieces in cell isolation buffer supplemented with 1 mg/mL bovine serum albumin (Sigma-Aldrich, Burlington, MA, US) and gentle shaking was applied to further facilitate dissociation of the tissue. The cell suspension was left to settle, and the cell pellet was resuspended in Tyrode buffer, containing (in mM): 133 NaCl, 4.8 KCl, 1.2 MgCl_2_, 10 glucose and 10 HEPES (pH 7.4; NaOH). Over the course of 20 minutes, the CaCl_2_concentration of the cell suspension was gradually increased to 0.6 mM.

During patch-clamp experiments, cardiomyocytes were superfused with Tyrode buffer containing 1.8 mM CaCl_2_ at 36 ± 0.5 °C. Patch-clamp data acquisition and analysis were performed as previously described [1] using a Multiclamp700B amplifier in conjunction with pClamp10.0 and a DigiData 1440A AD/DA interface (Molecular Devices, San Jose, CA, US). A pipette solution containing (in mM): 130 potassium aspartate, 0.1 Na-GTP, 5 Na_2_-AT, 11 EGTA, 5 CaCl_2_, 2 MgCl_2_, 10 HEPES (pH 7.2; KOH). Whole-cell intracellular access was obtained via a ruptured patch configuration. Action potentials (APs) were elicited by short (3 ms) blue light (470 nm) pulses at a frequency of 1 Hz. Optical stimulation was performed using a light emitting diode (LED, SPECTRA X light engine, Lumencor, Beaverton, OR, US) and a 20× objective (NA; 0.5, HCX PL FLUOTAR, Leica Microsystems, Wetzlar, DE), centred at wavelengths of 470 nm (6 mW/mm^2^) and 590 nm (7 mW/mm^2^). Light intensities (LIs) were measured at the sample site using a photodiode sensor (PD300-3 W, Ophir Optronics, Jerusalem, IL).

## Optical mapping: heart preparation and dye loading

The excised heart was immediately bathed in Krebs-Henseleit (KH) solution containing (in mM): 120 NaCl, 5 KCl, 2 MgS_2_ O_4_–7H_2_O, 20 NaHCO_3_, 1.2 NaH_2_PO_4_–H_2_O, 1.8 CaCl_2_ and 10 glucose (pH 7.4 when equilibrated with 95% oxygen and 5% carbon dioxide).

The heart was then cannulated through the aorta and retrogradely perfused with KH buffer containing 2 μM (±)-Blebbistatin (Enzo Life Sciences, Farmingdale, NY, US) to inhibit contraction. The cannulated heart was then placed in a custom-built optical mapping chamber with a horizontal Langendorff perfusion system where the heart was bathed and perfused with blebbistatin-containing KH solution at a constant flow of 2.5 mL/min at 36°C ± 0.5°C. Two platinum electrodes below the heart were used for monitoring cardiac electrical activity via electrocardiogram (ECG). 1 mL of perfusion solution containing the voltage sensitive dye (VSD) ElectroFluor 730p 8 μg/mL (University of Connecticut Health Center, Farmington, CT, US), was bolus injected into the cannula. Next, 50 µg of the calcium indicator X-Rhod-1, AM (Invitrogen, Thermo Fisher, Waltham, MA, US) was dissolved in 20% pluronic in DMSO (Invitrogen) and then diluted in 1 mL perfusion solution. The X-Rhod-1 loading solution was then slowly administered to the heart, injecting over a time course of 5 minutes.

All the experiments were performed within 1 hour after dye loading to avoid potential re-distribution of the dye and accumulation of phototoxic by-products.

## All-optical imaging and manipulation platform

Optical mapping and control were performed using a custom-made mesoscope which was previously described in detail [3]. Excitation light was provided by a light emitted diode (LED) and focussed on the heart in a wide-field configuration using a 2× objective (TL2x-SAP, Thorlabs, Newton, NJ, US). A 20× objective (LD Plan-Neofluar 20×/0.4 M27, Carl Zeiss Microscopy, Oberkochen, DE) was used to focus emission light on the central portion (128 × 128 pixels) of the sensor of a sCMOS camera (OrcaFLASH 4.0, Hamamatsu Photonics, Shizuoka, JP) operating at a frame rate of 1 kHz (1 ms actual exposure time). The detection path allows a field of view (at the object space) of 10.1 × 10.1 mm sampled with a pixel size of 80 μm.

For voltage imaging, a LED operating at a wavelength centred at 730 nm (M730L5, Thorlabs, Newton, NJ, US; maximum intensity of 1.066 mW/mm^2^) was used in combination with an excitation light filter centred at 730 nm (730/39 BrightLine HC, Semrock, Rochester, NY, US), a longpass dichroic mirror with edge at 757 nm (FF757-Di01, Semrock), and a emission light filter centred at 792 nm (792/64 BrightLine HC, Semrock). For calcium imaging, we used a LED emitting a wavelength centred at 590 nm (M590L4, Thorlabs; maximum intensity of 0.385 mW/mm^2^) in combination with an excitation light filter centred at 589 nm (589/15 BrightLine HC, Semrock), longpass dichroic mirror with edge at 605 nm (FF605-Di02, Semrock), and emission light filter centred at 630 nm (630/38 BrightLine HC, Semrock).

Optogenetic stimulation was performed by a Lightcrafter 4500 projector (Texas Instruments, Dallas, TX, US; maximum intensity of 4.26 mW/mm^2^), operating at a wavelength of ±470 nm.

Optogenetic pacing was performed by applying a pulse of 3 ms in the apical region of the heart. To illuminate in a sub-threshold fashion, the threshold for AP initiation was determined for each mouse heart, and illumination was applied a light intensity just below the threshold. The central portion of the heart was illuminated with a mean light intensity of 0.154 ± 0.036 mW/mm^2^. Hearts were electrically paced at 5, 6, and 8 Hz by an isolated constant-voltage stimulator (DS2A, Digitimer, Welwyn Garden City, Hertfordshire, UK), connected to the apex by a bipolar electrode. Light intensities were measured at sample site using a photodiode sensor (PD300-3W, Ophir Optronics, Jerusalem, IL).

## Optical mapping data and image analysis

All programs for data acquisition and analysis were developed with LabVIEW software (National Instruments, Austin, TX, US). For optical recordings, ΔF/F0 imaging was performed by processing raw data: for each frame, the background was first subtracted, then the voltage/calcium-independent bleaching effect in each individual trace was corrected by normalising the trace using either a linear or a non-linear polynomial fit applied to the diastolic phase. For each heart, AP and cytosolic Ca^2+^ kinetic properties were measured, trace by trace, to get the mean values after averaging ~5 subsequent systolic-diastolic cycles. AP maximum rising slope (APRS), AP duration (APD) at 50% and 90% of repolarisation (APD_50_, APD_90_ respectively), and Ca^2+^ maximum rising slope, calcium transient duration (CaD) at 50% and 90% of recovery (CaD_50_, CaD_90_) were measured in a central region of interest (ROI) of 50 × 50 pixels (≈ 25 mm^2^).

## Data presentation and statistics

Data is plotted as mean ± standard error of mean (SEM). Plots and statistical analyses were performed in GraphPad Prism software (version 9, GraphPad Software, San Diego, CA, US) Normality of data was confirmed by a Shapiro-Wilk test. The impact of 520 nm illumination on *V*_rest_ was assessed by a one-way repeated measures (RM) analysis of variance (ANOVA) test. For sub-threshold illumination experiments, two-way RM ANOVA tests with Tukey’s post hoc analysis was performed to assess the impact of optogenetic stimulation and pacing frequency.

# References

1. Coppini R, Mazzoni L, Ferrantini C, Gentile F, Pioner JM, Laurino A, Santini L, Bargelli V, Rotellini M, Bartolucci G, Crocini C, Sacconi L, Tesi C, Belardinelli L, Tardiff J, Mugelli A, Olivotto I, Cerbai E, Poggesi C (2017) Ranolazine Prevents Phenotype Development in a Mouse Model of Hypertrophic Cardiomyopathy. Circulation Heart failure 10:e003565. doi: 10.1161/CIRCHEARTFAILURE.116.003565

2. Scardigli M, Crocini C, Ferrantini C, Gabbrielli T, Silvestri L, Coppini R, Tesi C, Rog-Zielinska EA, Kohl P, Cerbai E, Poggesi C, Pavone FS, Sacconi L (2017) Quantitative assessment of passive electrical properties of the cardiac T-tubular system by FRAP microscopy. Proceedings of the National Academy of Sciences 114:5737–5742. doi: 10.1073/pnas.1702188114

3. Scardigli M, Müllenbroich C, Margoni E, Cannazzaro S, Crocini C, Ferrantini C, Coppini R, Yan P, Loew LM, Campione M, Bocchi L, Giulietti D, Cerbai E, Poggesi C, Bub G, Pavone FS, Sacconi L (2018) Real-time optical manipulation of cardiac conduction in intact hearts. The Journal of Physiology 596:3841–3858. doi: 10.1113/JP276283

4. Zaglia T, Pianca N, Borile G, Da Broi F, Richter C, Campione M, Lehnart SE, Luther S, Corrado D, Miquerol L, Mongillo M (2015) Optogenetic determination of the myocardial requirements for extrasystoles by cell type-specific targeting of ChannelRhodopsin-2. Proceedings of the National Academy of Sciences 112:E4495–E4504. doi: 10.1073/pnas.1509380112
